# Body Mass Index mediates the associations between dietary approaches to stop hypertension and obstructive sleep apnea among U.S. adults

**DOI:** 10.3389/fnut.2024.1509711

**Published:** 2024-12-16

**Authors:** Songtao Li, Yuxin Yang, Mengying Lin, Tian Lv, Yourang Pan, Jie Zhou

**Affiliations:** ^1^Department of Psychiatry, Shaoxing Seventh People’s Hospital, Affiliated Mental Health Center, Medical College of Shaoxing University, Shaoxing, China; ^2^College of Medical, Shaoxing University, Shaoxing, China; ^3^Department of Psychosomatic, Shaoxing Seventh People’s Hospital, Affiliated Mental Health Center, Medical College of Shaoxing University, Shaoxing, China; ^4^Department of Neurology, Zhuji Affiliated Hospital of Wenzhou Medical University, Zhuji, China

**Keywords:** OSA (obstructive sleep apnea), DASH (dietary approaches to stop hypertension), NHANES (National Health and Nutrition Examination Survey), BMI - Body Mass Index, mediation

## Abstract

**Background:**

The Dietary Approaches to Stop Hypertension (DASH) are associated with reduced cardiovascular, diabetes risk, but the effect on obstructive sleep apnea (OSA) is uncertain.

**Methods:**

This study used data from the National Health and Nutrition Examination Survey (NHANES). DASH score was assessed through 24-h dietary recall interviews, and OSA diagnosis in individuals was based on predefined criteria. Logistic regression analysis was used to assess the association between DASH and OSA. Restricted cubic spline (RCS) analysis was used to investigate the dose–response relationship between DASH score and OSA risk. And comprehensive subgroup and mediation analyses were performed.

**Results:**

Among the 14,978 participants, 27.01% had OSA. DASH scores had a negative association with the risk of OSA (OR = 0.91, 95%CI: 0.88–0.95, *p* < 0.01). Next, we divided DASH scores into quintiles groups. In comparison to the reference group Q1, groups Q5 had adjusted OR values of 0.63 (95%CI: 0.52–0.76, *p* < 0.01). Subgroup analyses revealed that this association was consistent across different groups. Further mediation analyses showed that the associations of DASH with OSA risk parallelly mediated by the above Body Mass Index (BMI) 33.4%,95%CI (20.6–46.2%) (all *p* < 0.05). The restricted cubic spline (RCS) analysis indicated a significant dose–response relationship between DASH diet and OSA risk.

**Conclusion:**

These findings suggested that DASH decreased OSA risk, which was possibly and partly mediated by BMI.

## Introduction

Obstructive sleep apnea (OSA) is a medical condition marked by repeated episodes of reduced or paused breathing during sleep, resulting in intermittent low oxygen levels, elevated carbon dioxide, and disturbed sleep (1). Common symptoms include snoring, breathing pauses, and reduced alertness during the day (2). Approximately 1 billion people globally are affected by OSA (3). OSA is recognized as an independent risk factor for hypertension, coronary artery disease, and stroke, and it has a strong link with insulin resistance and diabetes (4, 5). Obesity is the leading risk factor for OSA, while genetic predisposition, anatomical variations in the upper airway, and lifestyle choices like smoking and heavy alcohol use also contribute (6). A balanced diet can mitigate OSA symptoms by supporting weight management and providing benefits such as improved metabolism and reduced inflammation (7).

The DASH diet (Dietary Approaches to Stop Hypertension) focuses on a nutrient-dense approach, including abundant vegetables, fruits, whole grains, and low-fat dairy, while minimizing sodium, saturated fats, and cholesterol. It promotes higher intake of potassium, calcium, and magnesium-rich foods. Research indicates that a healthy diet can improve overall well-being by managing weight and metabolic health, and may also help prevent and manage OSA (8, 9). The DASH diet is recognized for its effectiveness in lowering blood pressure, improving cholesterol levels, and reducing chronic inflammation, making it a potential strategy for targeting OSA’s underlying causes. Specifically, its ability to lower blood pressure is key to reducing cardiovascular risks in individuals with OSA.

Hypertension and OSA are closely linked, with hypertension frequently occurring alongside OSA and potentially worsening its symptoms through mechanisms like vascular dysfunction and inflammation (10). The DASH diet, rich in antioxidants and fiber, has been proven to reduce oxidative stress and systemic inflammation, both of which play key roles in the development of OSA (11). Moran et al. found that the DASH diet helps reduce upper airway fat accumulation and decreases airway resistance through weight loss (12). Although the impact of diet on weight management, blood pressure control, and metabolic health is well-established, studies specifically examining its influence on OSA risk are limited. The DASH diet, known for its efficacy in reducing hypertension, is uniquely characterized by its emphasis on key nutrients such as potassium, calcium, magnesium, and low sodium. Recent evidence suggests that this dietary pattern also possesses anti-inflammatory properties and improves metabolic profiles (13).

This study aims to systematically assess how the DASH diet impacts OSA risk, considering BMI as a potential mediator, using the NHANES database, which provides a nationally representative sample of U.S. adults. The large sample size and diverse population enhance the generalizability of our findings, addressing a critical gap in understanding non-pharmacological strategies for OSA management.

## Methods

### Study design and participants

The NHANES is an ongoing survey conducted by the National Center for Health Statistics (NCHS), under the Centers for Disease Control and Prevention (CDC), aimed at assessing the health and nutritional status of the U.S. population. It collects data on demographics, physical examinations, laboratory tests, and dietary habits. Detailed information on survey design, data collection methods, and access to data files is available at http://www.cdc.gov/nchs/nhanes.html. Participants in NHANES provided written informed consent, with study protocols approved by the NCHS Research Ethics Review Board (14).

**Figure 1:**
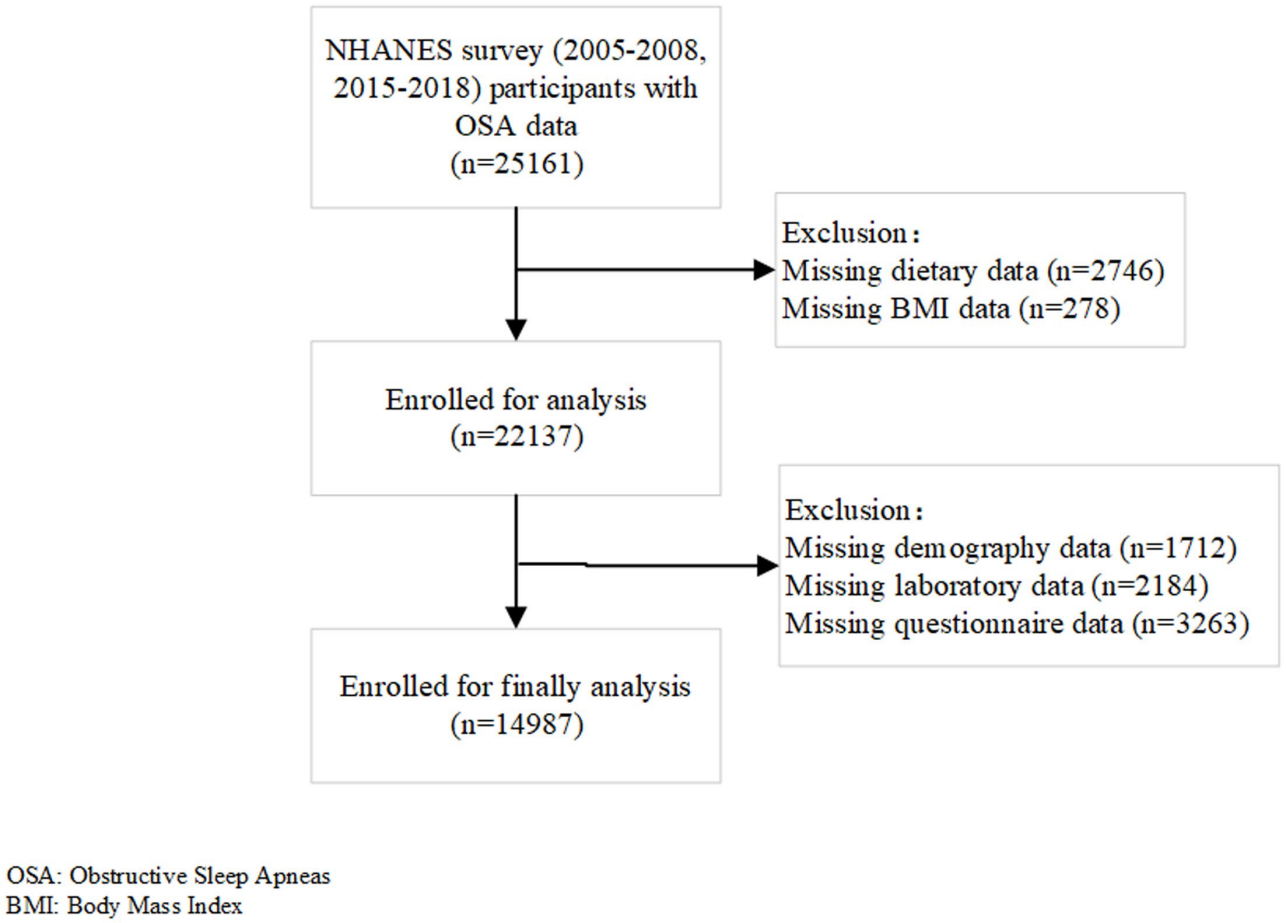
Flow chart of the study population.

The initial sample for this study included 25,161 participants aged 20 years and older, who had completed the sleep questionnaire during the 2005–2008 and 2015–2018 cycles. After excluding participants with missing data for dietary information (*n* = 2,746), BMI (*n* = 278), cotinine and systemic immune inflammation index (*n* = 1,080), waist circumference (*n* = 480), family income-to-poverty ratio (*n* = 1,780), albumin levels (*n* = 241), estimated Glomerular Filtration Rate (*n* = 1), smoking status (n = 1,849), alcohol consumption (*n* = 1,413), laboratory examination results (*n* = 382), hypertension status (*n* = 1), and education level (*n* = 4), the final analysis included 14,978 participants (Figure 1).

### Dietary information

Two 24-h dietary recall interviews were conducted to gather data on participants’ dietary intake. The first recall interview took place face-to-face, while the second was conducted via phone between 3 and 10 days after the initial session. The U.S. Department of Agriculture’s Food and Nutrient Database for Dietary Studies (FNDDS) was used to determine the energy and nutrient content of the reported food items (15). Average nutrient intake values were derived from the two recall interviews. The primary aim of these interviews was to collect comprehensive dietary intake data from participants. The collected data provided estimates of the types and amounts of foods and drinks (including all kinds of water) consumed during the 24-h period preceding the interview, and enabled the calculation of the energy, nutrients, and other food components contained in these items.

The DASH diet score for each participant was calculated based on their intake levels of foods and nutrients that are either emphasized or restricted by the DASH diet. This score focuses on nine specific nutrients: saturated fat, total fat, protein, cholesterol, fiber, magnesium, calcium, potassium, and sodium (16). Participants earned 1 point for meeting the set goal for a nutrient, 0.5 points for achieving an intermediate goal, and the score could reach a maximum of 9 points (17). Higher intakes of protein, fiber, magnesium, calcium, and potassium increased the score, while lower intakes of saturated fat, total fat, cholesterol, and sodium also contributed positively to the score. The scoring methodology is summarized in Supplementary Table S1.

### Definition of OSA

OSA symptoms were identified based on answers to three binary questions (18): (1) How often do you snore?; (2) How often do you experience snoring with pauses in breathing?; and (3) How often do you feel overly sleepy during the day? Participants who indicated snoring 3 or more times per week, snoring with breathing pauses 3 or more times per week, and feeling excessively sleepy during the daytime 16–30 times per month were categorized as having OSA symptoms.

### Covariates

Based on their relevance to dietary patterns, OSA, and obesity indicators, the following factors were considered as confounders: age, gender, education level, race, smoking status, alcohol consumption, advanced lung cancer inflammation index (ALI), Family Income-to-Poverty Ratio (PIR), white blood cell count, estimated Glomerular Filtration Rate (eGFR), Body Mass Index (BMI), history of diseases (diabetes, hypertension, and hyperlipidemia) and year (2005–2008, 2015–2018). Education level was categorized as either below college or college and above, while race was classified into non-Hispanic White, non-Hispanic Black, Mexican American, or other races. ALI was calculated using BMI, albumin levels, and the neutrophil-to-lymphocyte ratio (NLR) with the formula: BMI (kg/m^2^) × albumin (g/dL) / NLR, where NLR is the ratio of neutrophils to lymphocytes (19). The PIR represents the ratio of family income to the poverty threshold. Data on white blood cell counts were extracted from the NHANES database. The eGFR was calculated using the Chronic Kidney Disease Epidemiology Collaboration (CKD-EPI) equation (20). BMI was computed as weight (kg) divided by height squared (m^2^). Diabetes was defined according to American Diabetes Association (ADA) criteria and self-reported questionnaires. Participants were considered diabetic if they met any of the following: (1) fasting blood glucose (FBS) ≥7 mmol/L, (2) hemoglobin A1c ≥6.5%, (3) 2-h blood glucose ≥11.1 mmol/L during an oral glucose tolerance test, or (4) a self-reported diagnosis of diabetes and current use of insulin or other glucose-lowering medications (21). Hypertension was defined as having an average systolic blood pressure (SBP) ≥140 mmHg, an average diastolic blood pressure (DBP) ≥90 mmHg, or a self-reported diagnosis of hypertension (22). Hyperlipidemia was defined by total cholesterol ≥200 mg/dL, LDL cholesterol ≥130 mg/dL, HDL <40 mg/dL for men or < 50 mg/dL for women, triglycerides ≥150 mg/dL, or current use of lipid-lowering medications (23).

### Statistical analysis

Continuous variables were presented as means with standard deviations, while categorical variables were shown as frequencies and percentages. A *t*-test was used for comparisons of continuous variables, and chi-square tests were utilized for categorical variables. During descriptive analysis, continuous variables were reported as means with standard errors (SE), and categorical variables were displayed as weighted percentages (%). To assess the relationship between DASH scores and OSA, univariate and multivariate logistic regression models were used. Odds ratios (OR) were calculated to determine the strength of the association between DASH scores and OSA symptoms, with 95% confidence intervals (CI) provided for each estimate. The crude model did not include any adjustments for confounding factors. Model 1 adjusted for age, sex, education level, and race, while Model 2 included further adjustments for smoking status, alcohol consumption, ALI, PIR, WBC, BMI, eGFR, year cycle and comorbidities like diabetes, hypertension, and hyperlipidemia, based on Model 1 adjustments. To evaluate if a non-linear relationship between diet scores and OSA risk was present, restricted cubic spline (RCS) curves were used. If non-linearity was detected, a two-piece linear regression model was applied to determine the threshold effects of the diet score (24). To explore the potential mediator (BMI) between DASH score and OSA, a mediator analysis was conducted. This involved estimating the overall effect of DASH score on OSA (*α*), the effect of DASH score on BMI (β1), and the effect of the BMI on OSA (β2). The direct impact of DASH score on OSA was calculated as α - β1*β2 (25). Subgroup analysis aimed to assess potential effect modifications in the association between DASH scores and OSA, using stratification by age (<60 and ≥ 60), sex, BMI (<30 and ≥ 30), diabetes, hypertension, hyperlipidemia and year cycle. Stratified logistic regression models were used, and likelihood ratio tests assessed differences and interactions across subgroups. Adjustments for each subgroup followed Model 2.

The analyses were performed using R software version 4.2.1 (http://www.R-project.org, R Foundation). Statistical significance was defined as a two-sided *p*-value <0.05.

## Results

### Baseline characteristics

The baseline characteristics of the participants revealed significant differences between the OSA and non-OSA groups. Participants with OSA were older and had a higher prevalence of males (54.93%). Clinically, the OSA group exhibited higher BMI, elevated WBC counts, and lower eGFR. Lifestyle factors such as smoking and alcohol use were more prevalent in the OSA group, with higher rates of current smoking (24.10%) and heavy drinking (23.72%). Comorbidities, including hypertension, hyperlipidemia, and diabetes mellitus, were also significantly more common in the OSA group. Temporal trends indicated a higher proportion of OSA cases in the 2015–2018 cycle compared to 2005–2008. Dietary patterns showed that the OSA group had a lower mean DASH score and a higher proportion of individuals in the lowest DASH quintile (all p<0.05) (Table 1).

**Table 1:** Descriptive baseline characteristics of participants.

Variable	Total (*n* = 14,978)	Non-OSA (*n* = 10,524)	OSA (*n* = 4,454)	*p* value
Age	46.765(0.360)	46.050(0.374)	48.425(0.484)	< 0.0001
Gender				< 0.0001
Female	7,439(51.239)	5,446(53.899)	1993(45.066)	
Male	7,539(48.761)	5,078(46.101)	2,461(54.934)	
Education				0.108
College	7,735(60.396)	5,412(61.111)	2,323(58.736)	
Non-college	7,243(39.604)	5,112(38.889)	2,131(41.264)	
Race				0.576
Mexican American	2,551(8.433)	1817(8.674)	734(7.873)	
Non-Hispanic Black	3,067(10.094)	2,163(10.046)	904(10.205)	
Non-Hispanic White	6,587(69.502)	4,579(69.182)	2008(70.246)	
Other Hispanic	1,345(4.781)	942(4.822)	403(4.685)	
Other Race	1,428(7.191)	1,023(7.277)	405(6.991)	
PIR	3.105(0.044)	3.102(0.047)	3.111(0.058)	0.867
BMI (kg/m^2^)	29.024(0.125)	28.088(0.141)	31.196(0.160)	< 0.0001
WBC (1,000 cells/ul)	7.378(0.040)	7.291(0.045)	7.578(0.051)	< 0.0001
Lymphocyte (%)	30.512(0.133)	30.663(0.157)	30.163(0.209)	0.046
Monocyte (%)	8.045(0.032)	8.029(0.030)	8.083(0.062)	0.377
ALI	694.706(5.331)	681.101(6.367)	726.283(8.424)	< 0.0001
NLR	2.134(0.015)	2.120(0.017)	2.167(0.028)	0.155
SII	550.531(4.489)	547.903(5.005)	556.631(7.189)	0.269
eGFR (CKD-EPI)	95.026(0.532)	95.685(0.557)	93.499(0.684)	< 0.001
Smoking status				< 0.0001
Former	3,652(24.499)	2,454(23.424)	1,198(26.993)	
Never	8,242(54.791)	6,061(57.326)	2,181(48.909)	
Now	3,084(20.710)	2009(19.250)	1,075(24.097)	
Alcohol status				< 0.0001
Former	2,258(12.055)	1,553(11.276)	705(13.862)	
Heavy	3,030(22.054)	2072(21.339)	958(23.715)	
Mild	5,141(37.251)	3,538(37.295)	1,603(37.147)	
Moderate	2,417(18.064)	1,692(18.248)	725(17.636)	
Never	2,132(10.576)	1,669(11.842)	463(7.640)	
Hyperlipidemia				< 0.0001
No	4,414(29.704)	3,328(31.877)	1,086(24.660)	
Yes	10,564(70.296)	7,196(68.123)	3,368(75.340)	
Diabetes Mellitus				< 0.0001
Diabetes Mellitus	2,699(13.591)	1,648(11.304)	1,051(18.900)	
No	10,877(77.884)	7,897(80.470)	2,980(71.883)	
PRE-Diabetes Mellitus	1,402(8.524)	979(8.226)	423(9.216)	
Hypertension				< 0.0001
No	8,761(63.132)	6,500(66.897)	2,261(54.394)	
Yes	6,217(36.868)	4,024(33.103)	2,193(45.606)	
Year				< 0.0001
2005–2008	7,790(48.943)	5,679(50.777)	2,111(44.684)	
2015–2018	7,188(51.057)	4,845(49.223)	2,343(55.316)	
DASH score(0–9)	2.331(0.022)	2.392(0.025)	2.189(0.035)	< 0.0001
DASHQ				< 0.0001
Q1	4,099(26.695)	2,754(25.594)	1,345(29.252)	
Q2	2,163(14.486)	1,522(14.337)	641(14.831)	
Q3	3,547(24.340)	2,463(23.828)	1,084(25.528)	
Q4	2,518(17.142)	1809(17.302)	709(16.772)	
Q5	2,651(17.337)	1976(18.939)	675(13.617)	

OSA, Obstructive Sleep Apneas; PIR, Family Income-to-poverty Ratio; BMI, Body Mass Index; WBC, White blood cell; ALI, Advanced Lung cancer Inflammation index; NLR, Neutrophil–Lymphocyte Ratio; SII, Systemic Immune Inflammation Index; eGFR, estimated Glomerular Filtration Rate; CKD-EPI, Chronic Kidney Disease Epidemiology Collaboration; DASH, Dietary Approaches to Stop Hypertension; DASHQ, DASH score for quintiles.

Analysis of dietary data revealed that individuals with OSA often exhibited dietary patterns with higher energy intake, including increased consumption of fats, proteins, cholesterol, and sodium, alongside reduced intake of dietary fiber. These differences were statistically significant (*p* < 0.05) (Supplementary Table S2).

### Association between DASH scores and OSA

Table 2 presents the adjusted correlations between DASH scores and OSA. The results indicate a significant association between the DASH score and the incidence of OSA (Crude model: OR = 0.91, 95% CI: 0.88–0.95, *p* < 0.001; Model 1: OR = 0.92, 95% CI: 0.88–0.95, *p* < 0.001; Model 2: OR = 0.95, 95% CI: 0.92–0.99, *p* = 0.02). When DASH scores were divided into quintiles, participants in the highest quintile (Q5) showed a significantly lower risk of OSA compared to those in the lowest quintile (Q1, reference group). The odds ratios (ORs) for Q5 were: Crude model (OR = 0.63, 95% CI: 0.52–0.76, *p* < 0.001), Model 1 (OR = 0.64, 95% CI: 0.53–0.78, *p* < 0.001), and Model 2 (OR = 0.76, 95% CI: 0.62–0.94, *p* = 0.01) (Table 2).

**Table 2:** Multivariate logistic regression analyses for OSA and DASH score.

Exposure	Crude Model		Model 1		Model 2	
	95%CI	*P*	95%CI	*P*	95%CI	*P*
DASH score	0.91(0.88,0.95)	<0.0001	0.92(0.88,0.95)	<0.0001	0.95(0.92,0.99)	0.02
DASH score for quintiles						
Q1	ref		ref		ref	
Q2	0.91(0.76,1.08)	0.25	0.92(0.77,1.09)	0.34	0.97(0.81,1.17)	0.77
Q3	0.94(0.81,1.09)	0.39	0.96(0.83,1.11)	0.53	1.04(0.87,1.21)	0.65
Q4	0.85(0.71,1.01)	0.06	0.86(0.72,1.03)	0.10	0.95(0.80,1.14)	0.61
Q5	0.63(0.52,0.76)	<0.0001	0.64(0.53,0.78)	<0.0001	0.76(0.62,0.94)	0.01
p for trend		<0.0001		<0.0001		0.03

Crude Model: only DASH score. Model 1: adjusted for included crude model, age, gender, education and race. Model 2: adjusted for all Model 1in addition to smoking status, alcohol status, ALI, diabetes, hyperlipidemia, hypertension, PIR, WBC, BMI and year cycle.

**Figure 2:**
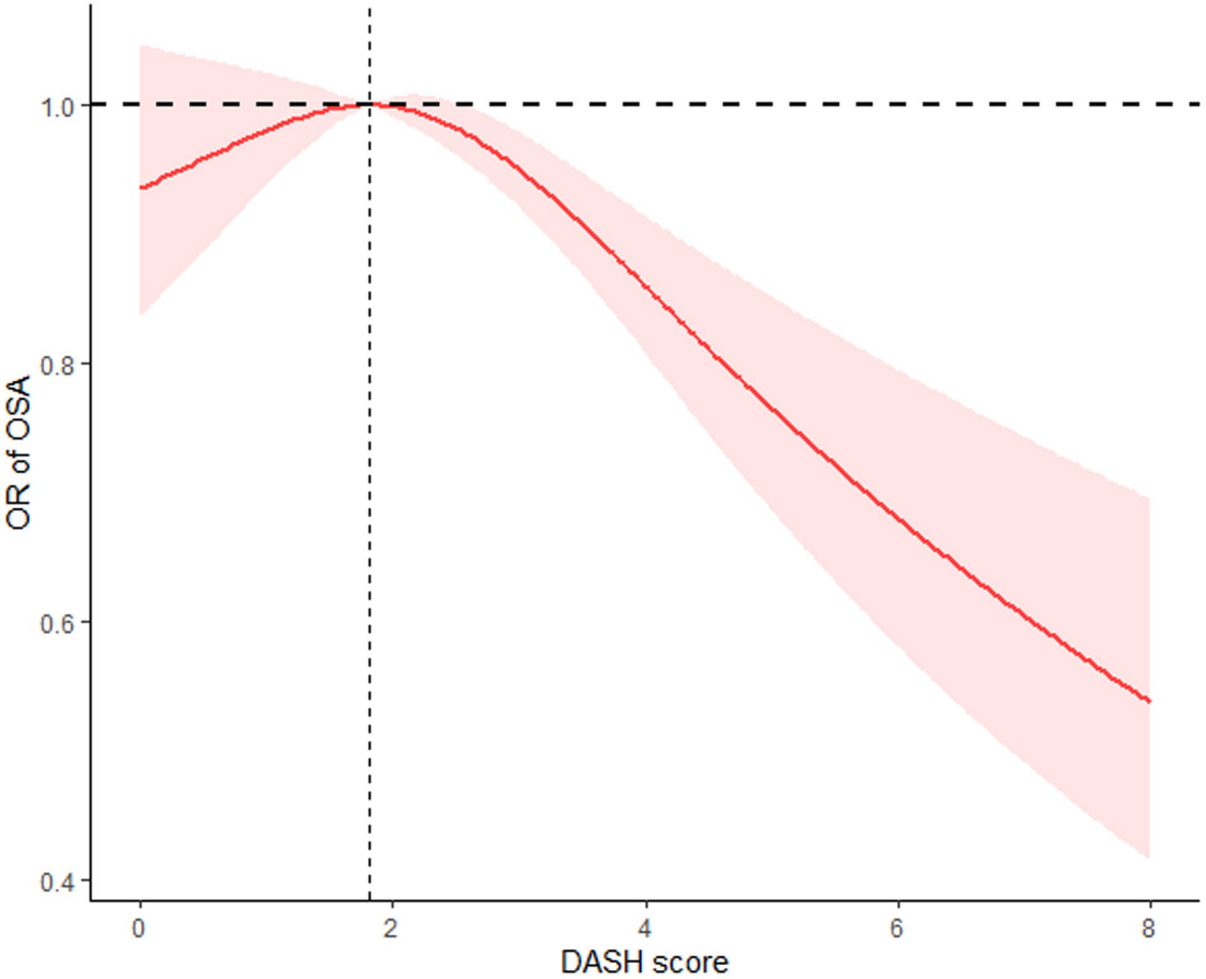
The restricted cubic spline (RCS) curve between DASH score and OSA incidence based on Model 2.

The restricted cubic spline analyses with multivariable adjustments revealed an inverse L-shaped relationship between the DASH score and OSA incidence. To provide a more detailed analysis of the relationship between DASH adherence and OSA risk, we conducted restricted cubic spline (RCS) analyses using both the crude model and Model 1. The crude model, which adjusts only for DASH adherence, showed an inverse L-shaped relationship (Supplementary Figure S1). When adjusted for gender, age, education level, and race in Model 1, the trend remained consistent but showed slightly adjusted effect estimates (Supplementary Figure S2). As for Model 2, the inflection point of the curve occurred at a DASH score of 1.809. A two-piece linear regression analysis showed that on the right side of the threshold, the odds ratio (OR) was 0.90 (95% CI: 0.86–0.96, *p* < 0.001), meaning that each 1-point increase in the DASH score was associated with a 10% reduction in OSA risk. In contrast, on the left side of the threshold, the OR was 1.04 (95% CI: 0.86–1.22, *p* = 0.63), indicating no statistically significant relationship between the DASH score and OSA incidence (Figure 2).

### Subgroup analysis

**Figure 3:**
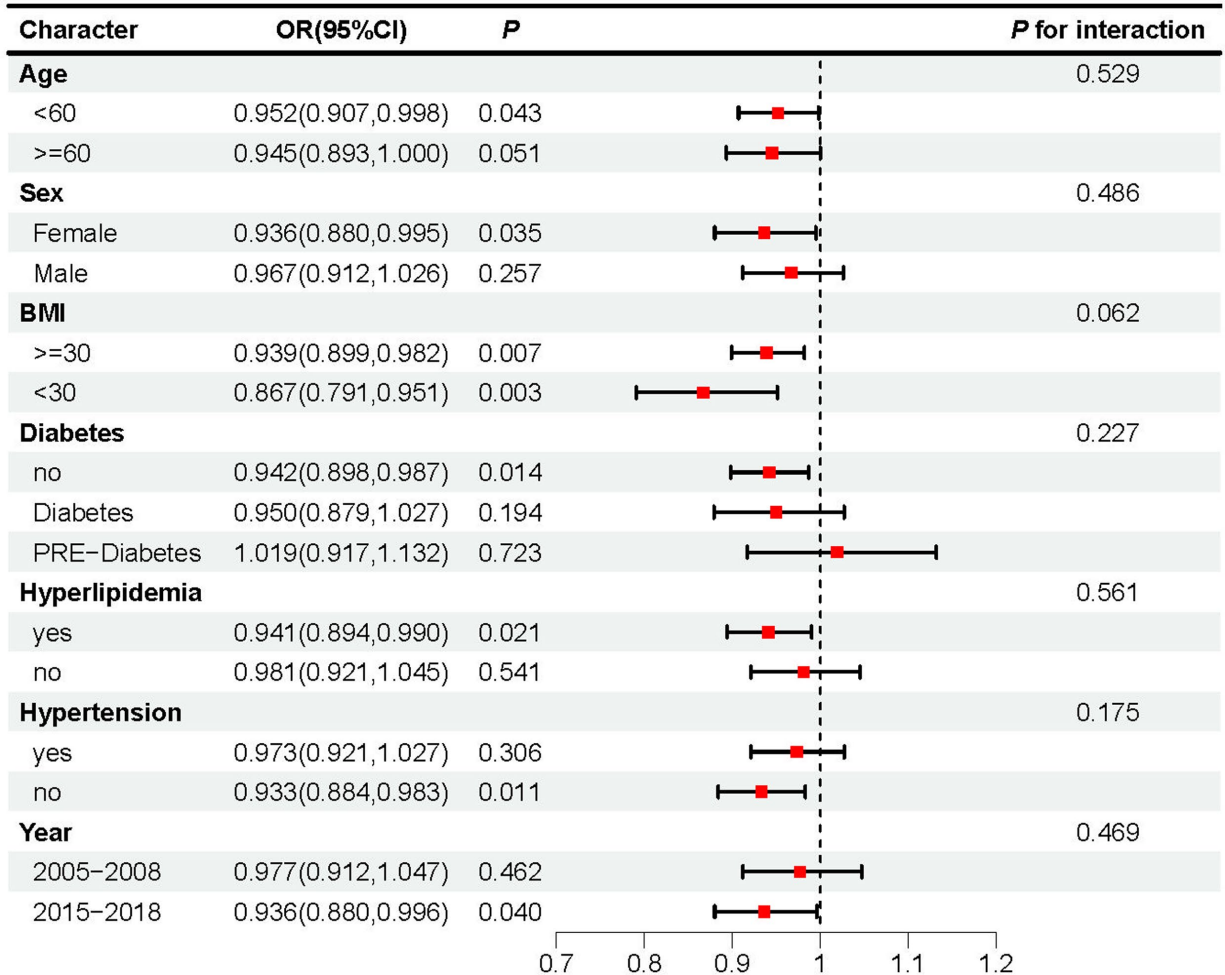
Subgroup analyses assessing the effect of DASH score on OSA incidence.

Subgroup analysis revealed that adherence to the DASH diet was linked to a lower risk of OSA in individuals under 60 years old (OR: 0.952, 95% CI: 0.907–0.998, *p* = 0.043), females (OR: 0.936, 95% CI: 0.880–0.995, *p* = 0.035), time span from 2015 to 2018 (OR: 0.936, 95%CI: 0.880–0.996, p = 0.04), those with hyperlipidemia (OR: 0.941, 95% CI: 0.894–0.990, *p* = 0.021), and individuals without hypertension (OR: 0.933, 95%CI: 0.884–0.983, *p* = 0.011) or without diabetes (OR: 0.942, 95% CI: 0.898–0.987, *p* = 0.014). Adherence to the DASH diet was also significantly associated with a reduced OSA risk, irrespective of BMI status: both in individuals with BMI ≥30 (OR: 0.939, 95% CI: 0.899–0.982, *p* = 0.007) and those with BMI <30 (OR: 0.867, 95% CI: 0.791–0.951, *p* = 0.003). Moreover, no significant differences were found between the subgroups (*p* > 0.05), indicating that the results of the subgroup analysis are consistent and reliable (Figure 3).

### Mediation analyses

**Figure 4:**
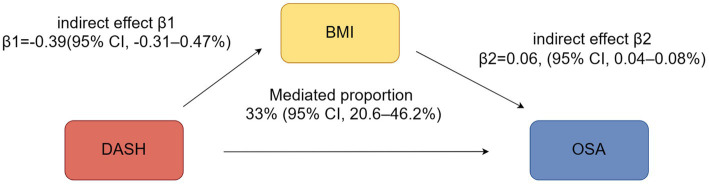
The mediating effect analysis of DASH Score and OSA.

A mediation analysis was performed to assess the role of BMI in the association between DASH scores and OSA risk. The analysis revealed that BMI significantly mediated this relationship, accounting for an indirect effect of 33.4% (95% CI: 20.6 to 46.2%, *p* < 0.05) (Figure 4).

## Discussion

This study revealed a significant inverse association between adherence to the DASH diet and OSA risk. Leveraging the NHANES database, which encompasses a large and representative sample of the U.S. adult population, our findings offer robust evidence supporting the role of dietary interventions in managing OSA risk. The inclusion of a diverse demographic enhances the clinical relevance and applicability of our results to public health strategies.

The DASH diet was originally designed to prevent and manage hypertension. It emphasizes increased intake of vegetables, fruits, whole grains, and low-fat dairy, while limiting sodium, saturated fats, and added sugars. This diet has been shown to effectively reduce blood pressure and improve cardiovascular health. Excessive sodium intake can cause water and sodium retention, increasing blood volume and exerting more pressure on blood vessel walls, which contributes to hypertension. Studies suggest that reducing sodium intake can significantly decrease blood pressure, particularly in those with existing hypertension (26). Additionally, nutrients such as potassium, calcium, and magnesium are crucial for regulating blood pressure. Potassium lowers blood pressure by aiding in sodium excretion and reducing blood vessel constriction (27). Calcium and magnesium contribute to blood pressure stability by promoting the relaxation of vascular smooth muscles and preventing blood vessel constriction (28). Beyond its cardiovascular and metabolic benefits, the DASH diet also supports improved endothelial function and insulin sensitivity (29, 30). Liu et al. (31) further discovered that the DASH diet, when combined with the Mediterranean diet, effectively reduces the risk of Alzheimer’s disease. This finding suggests that the DASH diet may play a role in the prevention of neurodegenerative diseases (31).

The link between metabolic syndrome and OSA is complex. Metabolic syndrome is characterized by conditions like diabetes, hypertension, dyslipidemia, and central obesity (32). These metabolic disturbances contribute to the development and progression of OSA through multiple mechanisms. Obesity, a major risk factor for OSA, leads to excess fat accumulation, especially in the abdominal and neck regions. This buildup around the upper airway increases the risk of airway collapse (33). This mechanical effect aggravates airway obstruction, leading to breathing difficulties and intermittent hypoxia, which in turn worsens insulin resistance and inflammation, forming a self-perpetuating cycle (34).

In addition to mechanical factors, metabolic syndrome accelerates the progression of OSA through systemic inflammation and oxidative stress. Studies indicate that metabolic imbalances can trigger a prolonged inflammatory response, impairing endothelial function, disrupting blood flow and microcirculation, and decreasing oxygen delivery (35). When oxidative stress is combined with chronic inflammation, it further diminishes upper airway function, worsening the severity of OSA (36). These pathways highlight the significant role of metabolic syndrome in OSA, indicating that managing factors like obesity and insulin resistance is crucial for alleviating OSA symptoms.

BMI serves as a crucial mediator in the relationship between metabolic syndrome and OSA, as higher BMI is strongly linked to increased OSA prevalence. Elevated BMI is associated with various components of metabolic syndrome, including hyperglycemia, hypertension, and dyslipidemia, all of which can exacerbate OSA symptoms (37). Therefore, BMI is not just a result of metabolic disturbances but also a critical factor in the development of OSA (38). BMI plays a significant mediating role in the relationship between DASH diet adherence and OSA risk, likely through multiple physiological pathways. One key mechanism is the reduction of fat accumulation, particularly in the upper airway, which decreases the likelihood of airway obstruction during sleep (39). Furthermore, lower BMI is associated with improved airway resistance, contributing to enhanced airflow and reduced apnea episodes (40). These findings underscore the importance of weight management as a crucial strategy for mitigating OSA risk. The weight gain and heightened metabolic burden due to obesity create a bidirectional feedback loop, where in OSA and metabolic syndrome mutually exacerbate, driving the progression of both conditions.

This study demonstrates that the DASH diet lowers OSA risk by improving metabolic syndrome, with a notably strong protective effect in individuals with hyperlipidemia and those without diabetes. It is proposed that components of the DASH diet may reduce OSA risk by influencing BMI. Our findings support this hypothesis, revealing that the DASH diet significantly reduces OSA risk through BMI reduction. Mediation analysis confirmed that BMI plays a significant mediating role between the DASH diet and OSA risk, highlighting its indirect protective effect. Subgroup analysis further revealed that higher DASH diet scores were associated with reduced OSA risk in both obese and non-obese groups, with no significant differences between them, underscoring the broad applicability and consistency of this protective effect.

Previous research suggests that the dietary fiber in the DASH diet can lower blood glucose levels and enhance insulin sensitivity, thereby reducing the risk of diabetes (41, 42). The DASH diet’s emphasis on limiting saturated fats and cholesterol, while favoring unsaturated fats, leads to improved lipid profiles (43). Moreover, the diet’s inclusion of antioxidant-rich fruits and vegetables is vital for minimizing oxidative stress and stabilizing blood glucose levels (44, 45). The RCS curve results suggest that adherence to the DASH diet begins to significantly reduce OSA risk when the DASH score exceeds 1.809. This highlights the clinical importance of achieving a DASH score of at least 2, as it marks the point where noticeable protective effects against OSA emerge. Targeted dietary strategies should focus on promoting adherence that reaches or exceeds this level to maximize health benefits. The results of the subgroup analyses for the two NHANES cycles (2005–2008 and 2015–2018) showed consistent associations between DASH adherence and OSA risk across both periods. The interaction *p*-value (0.469) indicated no statistically significant differences between the 2 cycles. These findings suggest that the relationship between DASH adherence and OSA risk is stable over time and is unlikely to be substantially influenced by temporal changes in dietary habits, OSA prevalence, or health status. Thus, to effectively lower blood lipids and glucose, reduce metabolic burden, improve BMI, and manage metabolic conditions through the DASH diet, strict adherence to its principles is crucial. Such comprehensive adherence is essential for realizing significant health benefits and enhancing overall well-being.

In conclusion, the DASH diet exerts a protective effect against OSA through multiple mechanisms, with BMI serving as a central mediator. This study’s findings offer further evidence supporting these mechanisms, emphasizing the DASH diet as an effective strategy for both preventing and managing OSA. Although previous research has primarily concentrated on the role of healthy diets in managing OSA-related conditions such as hypertension, cardiovascular disease, and diabetes (46), direct studies on the impact of diet on OSA risk are limited and often involve small sample sizes without support from large-scale data (47). Additionally, the dose–response relationship between diet and OSA risk is not yet fully understood, indicating a need for more research to refine the precision and efficacy of dietary interventions in clinical practice (48).

### Limitations

The cross-sectional design of this study limits its ability to assess the long-term effects of the DASH diet on OSA or to perform survival analysis, thus restricting its capacity to determine causal relationships. Dietary data were collected through a 24-h recall method, which may not accurately capture participants’ usual dietary habits over time. The assessment of 24-h dietary intake may not accurately capture an individual’s lifetime dietary patterns, raising questions about whether the reported intake truly represents their typical daily diet. Future studies, particularly longitudinal designs, are needed to better evaluate long-term adherence to dietary patterns and their impact on health outcomes. The DASH diet score used in this study reflects the dietary intake reported by participants over a 24-h period. While this scoring system provides valuable insights into dietary patterns, it does not directly measure participants’ long-term adherence or intent to follow the DASH diet. The assessment of 24-h dietary intake may not accurately capture an individual’s habitual diet or lifetime dietary patterns, which is a limitation of the study design. We have acknowledged in the revised manuscript that this study focuses specifically on the association between the DASH diet and OSA, while not accounting for other dietary patterns and their potential impact on OSA. This may limit our ability to fully reflect the dietary behaviors of the target population. Similarly, our study provides valuable initial insights but could benefit from comparisons with other dietary patterns in future research. Nonetheless, dietary intake assessments in NHANES have undergone thorough validation against dietary records and biomarker data (49). Additionally, the OSA diagnosis in this study relied primarily on self-reported questionnaires rather than objective assessments like polysomnography, potentially introducing bias, as symptom reporting may be influenced by participants’ subjective perceptions.

## Conclusion

This study explored the relationship between the DASH diet and the risk of OSA, revealing that individuals who adhered to DASH dietary recommendations had a lower risk of OSA. The significant role of BMI as a mediator and the identified dose–response relationship between the DASH diet and OSA offer practical implications for non-pharmacological intervention strategies. Further studies are needed to confirm the direct impact of the DASH diet on OSA management, which could provide the scientific foundation for dietary intervention guidelines and support the expanded clinical use of the DASH diet.

## Data Availability

The original contributions presented in the study are included in the article/Supplementary material, further inquiries can be directed to the corresponding authors.

## Supplementary material

SUPPLEMENTARY FIGURE S1 The restricted cubic spline (RCS) curve between DASH score and OSA incidence based on Crude Model.

SUPPLEMENTARY FIGURE S2 The restricted cubic spline (RCS) curve between DASH score and OSA incidence based on Model 1.
