# Supplementary figures and images for: Body Mass Index mediates the associations between dietary approaches to stop hypertension and obstructive sleep apnea among U.S. adults
Source: Front Nutr. 2024 Dec 16;11:1509711. doi: 10.3389/fnut.2024.1509711 (PMC11682964; doi:10.3389/fnut.2024.1509711)

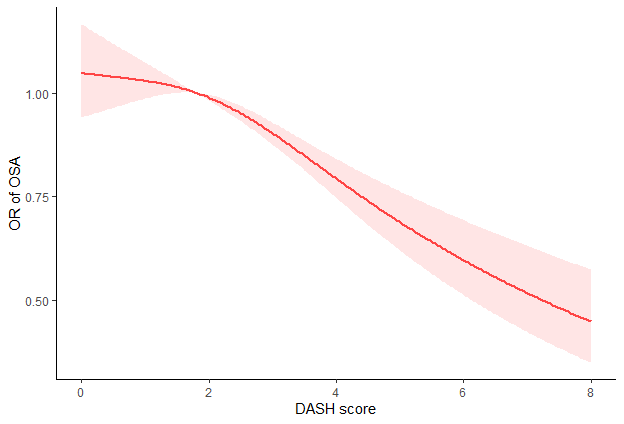

Supplement: SUPPLEMENTARY FIGURE S1 — The restricted cubic spline (RCS) curve between DASH score and OSA incidence based on Crude Model. [file Image_1.tiff]

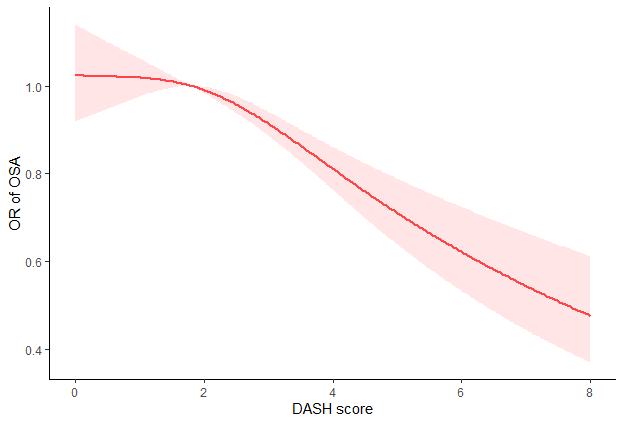

Supplement: SUPPLEMENTARY FIGURE S2 — The restricted cubic spline (RCS) curve between DASH score and OSA incidence based on Model 1. [file Image_2.tiff]
