# Supplementary material for: Body Mass Index mediates the associations between dietary approaches to stop hypertension and obstructive sleep apnea among U.S. adults
Source: Front Nutr. 2024 Dec 16;11:1509711. doi: 10.3389/fnut.2024.1509711 (PMC11682964; doi:10.3389/fnut.2024.1509711)
Supplement: Supplementary file 3 [file Table_1.DOCX]

Table S1 Nutrient Targets for DASH Score

| Nutrient | DASH Diet  Nutrient  Composition | DASH  Score Target | Intermediate  Target |
| --- | --- | --- | --- |
| Saturated fat | 6% of energy | 6% of energy | 11% of energy |
| Total fat | 27% of energy | 27% of energy | 32% of energy |
| Protein | 18% of energy | 18% of energy | 16.5% of energy |
| Cholesterol | 150 mg | 71.4 mg/1000 kcal | 107.1 mg/1000 kcal |
| Fiber | 31 g | 14.8 g/1000 kcal | 9.5 g/1000 kcal |
| Magnesium | 500 mg | 238 mg/1000 kcal | 158 mg/1000 kcal |
| Calcium | 1240 mg | 590 mg/1000 kcal | 402 mg/1000 kcal |
| Potassium | 4700 mg | 2238 mg/1000 kcal | 1534 mg/1000 kcal |
| Sodium | 2400 mg | 1143 mg/1000 kcal | 1286 mg/1000 kcal |
