# Supplementary material for: Body Mass Index mediates the associations between dietary approaches to stop hypertension and obstructive sleep apnea among U.S. adults
Source: Front Nutr. 2024 Dec 16;11:1509711. doi: 10.3389/fnut.2024.1509711 (PMC11682964; doi:10.3389/fnut.2024.1509711)
Supplement: Supplementary file 4 [file Table_2.DOCX]

Table S2 Description based on dietary data

| Variable | Total（n=14978） | Without OSA（n=10524） | With OSA（n=4454） | P value |
| --- | --- | --- | --- | --- |
| Saturated fat (g) | 28.308(0.270) | 27.680(0.301) | 29.766(0.429) | < 0.0001 |
| Total fat (g) | 85.715(0.706) | 83.758(0.816) | 90.259(1.094) | < 0.0001 |
| Protein (g) | 84.214(0.639) | 83.044(0.772) | 86.929(1.038) | 0.003 |
| Cholesterol (mg) | 302.988(2.921) | 294.671(3.766) | 322.293(5.821) | < 0.001 |
| Fiber (g) | 8.009(0.091) | 8.222(0.101) | 7.512(0.110) | < 0.0001 |
| Magnesium (mg) | 306.713(2.936) | 305.659(3.370) | 309.160(4.392) | 0.48 |
| Calcium (mg) | 966.131(10.087) | 963.978(11.076) | 971.129(18.202) | 0.721 |
| Potassium (mg) | 2688.345(21.738) | 2665.203(24.527) | 2742.061(37.398) | 0.07 |
| Sodium (mg) | 3574.462(26.540) | 3510.129(33.611) | 3723.787(39.142) | < 0.0001 |
| Energy (kcal) | 2174.294(13.494) | 2126.649(15.577) | 2284.883(21.326) | < 0.0001 |
